# A Novel Predictor for Micro-Scale COVID-19 Risk Modeling: An Empirical Study from a Spatiotemporal Perspective

**DOI:** 10.3390/ijerph182413294

**Published:** 2021-12-16

**Authors:** Sui Zhang, Minghao Wang, Zhao Yang, Baolei Zhang

**Affiliations:** College of Geography and Environment, Shandong Normal University, Jinan 250014, China; 201814010401@stu.sdnu.edu.cn (S.Z.); 201814010418@stu.sdnu.edu.cn (M.W.); 201814010402@stu.sdnu.edu.cn (Z.Y.)

**Keywords:** COVID-19, gravity model, geographically and temporally weighted regression (GTWR), spatiotemporal risk modeling, Qingdao

## Abstract

Risk assessments for COVID-19 are the basis for formulating prevention and control strategies, especially at the micro scale. In a previous risk assessment model, various “densities” were regarded as the decisive driving factors of COVID-19 in the spatial dimension (population density, facility density, trajectory density, etc.). However, this conclusion ignored the fact that the “densities” were actually an abstract reflection of the “contact” frequency, which is a more essential determinant of epidemic transmission and lacked any means of corresponding quantitative correction. In this study, based on the facility density (FD), which has often been used in traditional research, a novel micro-scale COVID-19 risk predictor, facility attractiveness (FA, which has a better ability to reflect “contact” frequency), was proposed for improving the gravity model in combination with the differences in regional population density and mobility levels of an age-hierarchical population. An empirical analysis based on spatiotemporal modeling was carried out using geographically and temporally weighted regression (GTWR) in the Qingdao metropolitan area during the first wave of the pandemic. The spatiotemporally nonstationary relationships between facility density (attractiveness) and micro-risk of COVID-19 were revealed in the modeling results. The new predictors showed that residential areas and health-care facilities had more reasonable impacts than traditional “densities”. Compared with the model constructed using FDs (0.5159), the global prediction ability (adjusted R^2^) of the FA model (0.5694) was increased by 10.4%. The improvement in the local-scale prediction ability was more significant, especially in high-risk areas (rate: 107.2%) and densely populated areas (rate in Shinan District: 64.4%; rate in Shibei District: 57.8%) during the outset period. It was proven that the optimized predictors were more suitable for use in spatiotemporal infection risk modeling in the initial stage of regional epidemics than traditional predictors. These findings can provide methodological references and model-optimized ideas for future micro-scale spatiotemporal infection modeling.

## 1. Introduction

The COVID-19 pandemic is the most serious global public health event that has occurred in the 21st century thus far and has become a hot topic in many different disciplines [1]. The global pandemic has severely damaged the global economy, society, finance, and even the ecosystem and environment [2,3,4,5], highlighting the importance of recognizing the “risks” of regional epidemics [6,7]. The level of risk not only shows the current situation of epidemic infection and the probability of new cases occurring in a region, but, more essentially, determines what level of prevention and control measures should be taken in this region to reduce the risk of a pandemic as far as possible [8,9]. At the same time, the rapid transmission of COVID-19 and its complexity mean that the time and space in which both human–human and human–place interactions take place are particularly important in the study of the spatial epidemiology of COVID-19 [10,11,12]. Therefore, it is self-evident that the quantitative analysis and micro-modeling of COVID-19 from the spatiotemporal perspective are necessary for epidemic risk measurement, especially in the initial stage before the complete outbreak of a regional epidemic [13,14]. 

Based on the empirical evidence provided by previous studies, people’s mobility and contact are considered as decisive factors in the transmission of COVID-19 [11,15]. Theoretically, the compact development of cities will lead to closer contact between people and more frequent interactions occurring among residents, which also makes these high-density areas potential hot spots for the rapid spread of emerging epidemics [16,17]. Therefore, “densities” are given priority in most cases when micro-modeling the spread and risk of infectious diseases [18,19]. “Densities” can be divided into three categories: population density, trajectory density, and facility density [11,20,21]. Population density has always been regarded as the determinant of epidemic exposure and transmission risk, and many studies have explored the complex relationship between population density and epidemic spread in various spatiotemporal contexts [22,23]. However, because most population density data are in the form of statistical data, corresponding high-resolution geospatial data usually need to undergo complex spatial correction processing, and it is difficult to carry out microscopic epidemiological modeling directly [24,25]. This has caused urban big data, which are more accurate than population data, to generally receive more attention in recent studies [26,27]. Density analyses based on trajectory data (smartphone data, traffic trajectory) have been implemented in many studies, providing unique advantages for the analysis of population dynamic characteristics and the initial modeling of epidemic diseases [11,28]. However, due to the high cost of accessing the trajectory data, some researchers use points of interest (POI) instead to combine and model specific or multiple facility densities. Different from urban roads, another spatial element of cities that often leads to people being more mobile is the closeness of different facilities. This causes more people to gather in one location and thus plays a pivotal role in the transmission of highly contagious epidemics. Complementing population density, which is commonly used in the classical theory of infectious disease ecology and transmission, the use of various facility densities can better simulate the people’s contact frequency. Due to these characteristics, modeling studies that predict epidemic risk from the perspective of urban planning based on facility density, the mapping of urban functions in gathering places, and calculating the probability of people gathering and coming into contact in airtight places have attracted great attention [21,29,30]. Although several empirical studies have highlighted the significance of the local epidemic source of COVID-19 as a driving mechanism in the initial stage of the pandemic, various density indicators play a dominant role in the modeling of micro-scale spatiotemporal epidemiology [13,31].

However, the use of “densities” is obviously not perfect for the micro-scale spatiotemporal modeling of COVID-19 risk. Firstly, a considerable number of studies have found that “densities” have limited effects in the initial stage of regional epidemics [31]. Secondly, no matter what the simple population density is, trajectory density or facility density cannot reflect the real mechanism of human interaction. Although they can simulate the static and dynamic density of the population (population density, track density) and the possibility of people gathering in one space (facility density), respectively, such one-sided modeling usually leads to biased estimations of densely populated areas with low gathering possibilities and sparsely populated areas with high possibilities of people gathering [16,17,32]. In addition, differences in the age structure of the population and resulting differences in travel modes and capacities are usually not considered in the micro-modeling of COVID-19 risk [13,33]. To sum up, although traditional “densities” models have been widely used in previous studies and achieved excellent results, compared with non-compound “densities” the deeper determinants of the intracity risks of epidemics are undoubtedly more multi-levelled. When complex population structures and the spatial heterogeneity of different types of facility density are considered, the result of comprehensive modeling is not so much related to “densities” in the traditional meaning, but more related to “contact”, which is similar to the word “interaction” (which is often mentioned in the field of geographical analysis) [34,35]. Therefore, in this study we try to simulate the frequency of human–human and human–place interactions occurring between different kinds of people and gathering places based on low-cost and accessible data. Taking population distribution, age structure, and gathering probability into account, a quantitative predictor, facility attractiveness (FA), which can be widely applied instead of the predictor of traditional facility density (FD), was used. We then built a spatiotemporal risk model in a small area where there was an outbreak of COVID-19 and evaluated its prediction efficiency in different application scenarios.

The main purposes of this study include: (1) improving the gravity model to build facility attractiveness (FA); (2) modeling and visualizing the varying spatiotemporal impact of the selected driving factors on the risk of COVID-19 in the Qingdao metropolitan area using geographically and temporally weighted regression (GTWR); (3) comparing and discussing the advantages and disadvantages of the traditional FD model and the novel FA model in different application scenarios.

## 2. Data and Methodology

### 2.1. Empirical Area

**Figure 1:**
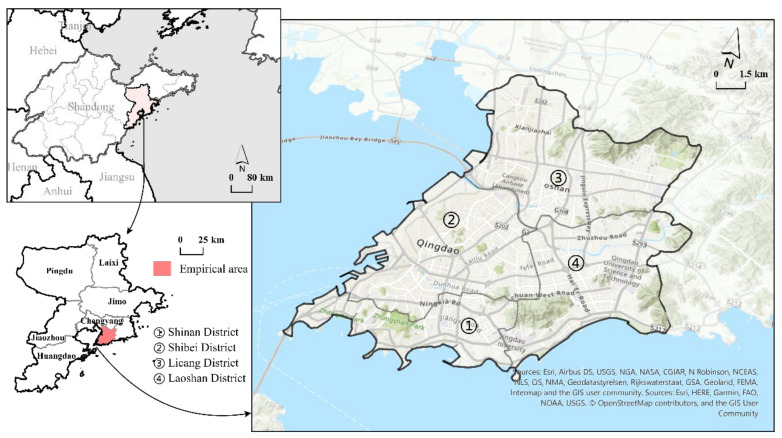
The geographical location of the empirical area.

Qingdao is located in the east of Shandong Province, China, facing Japan and South Korea across the sea. It is the economic center of Shandong Province and an important international port city in China. Based on its geographical location and important business status, Qingdao is considered to be one of the regions with the highest risk of the occurrence of a local epidemic in China. In this study, the Qingdao metropolitan area was selected as our empirical area (120°52′~120°28′ E, 36°204′~36°24′ N). This area is about 249.3 km^2^ and represents only 2.21% of Qingdao, but more than 42% of its population, making it a densely populated area within Qingdao and an area with a high COVID-19 infection risk (Figure 1). To better understand the spatiotemporal risk of COVID-19 at the micro scale, we referred to the idea of Ling (2020) regarding community grid management and considered the advantages of the use of a hexagonal grid for dealing with adjacency problems, modified area unit problems (MAUPs), and high-precision spatiotemporal modeling [31,36]. In our study, a hexagonal grid with a side length of 250 m was used to divide the study area into blocks, and 1684 independent hexagons were selected for further analysis [37,38]. 

### 2.2. Data Sources and Measures

**Figure 2:**
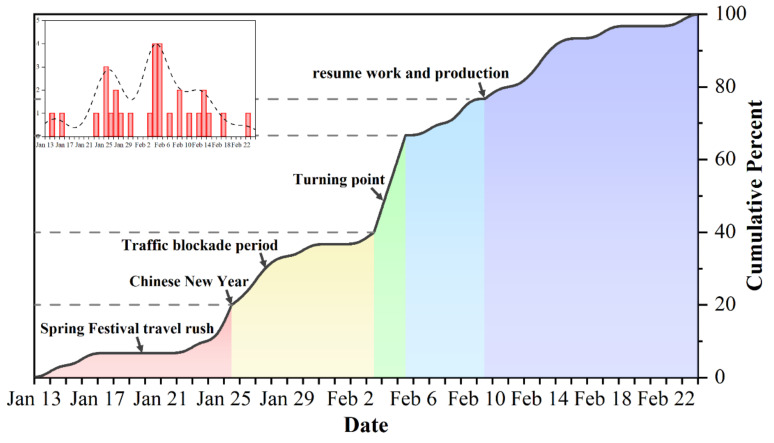
Development and stage definition of COVID-19 in the empirical area from 14 January to 23 February.

The data used in this study include: COVID-19 case data, POI data, OpenStreetMap road network data, and WorldPop population age structure data. Among these, the case data include information regarding the age, gender, address, and date of symptom onset of all cases confirmed in the empirical area from January to May, 2020, from the Qingdao Municipal Health Commission (wsjkw.qingdao.gov.cn, accessed on 1 June 2020). The POI data were sourced from Amap (www.amap.com, accessed on 6 June 2020). In this study, catering places, residential areas, shopping places, public service facilities, health-care facilities, education places, and entertainment places were initially highlighted as places where people gather. However, due to the strict lockdown measures implemented in China since January 2020, the possibility of people gathering in most education and entertainment venues in Qingdao has become negligible (Figure 2). Therefore, this study excluded these variables, and the remaining five types of places that experience the greatest amount of human activity and that represent the greatest possibility of disease transmission within the context of the pandemic were selected as independent variables (catering places, residential areas, shopping places, public service facilities, and health-care facilities). The 100 m-resolution raster data for people of various ages were sourced from WorldPop (www.worldpop.org, accessed on 1 March 2021); the road network data came from OpenStreetMap (www.openstreetmap.org, accessed on 1 June 2020). 

In order to construct effective and reliable panel data to meet the computational requirements of geographically and temporally weighted regression (GTWR) and according to the date of onset of these cases (Figure 2), the local epidemic was divided into five stages (Stage 1: 14 January to 25 January; Stage 2: 26 January to 3 February; Stage 3: 4 February to 5 February; Stage 4: 6 February to 10 February; Stage 5: 11 February to 23 February). Stage 1 represents the initial stage of the local epidemic; before the Chinese New Year, the end of this stage, many people in China were engaging in annual large-scale population migration, the so-called (pre-) Spring Festival travel rush, which enabled COVID-19 to spread rapidly across the country. Stage 2 represents the accelerate stage of the local epidemic, which was also the strictest traffic blockade period; after this stage, the traffic blockade of the Chinese mainland, except for Hubei Province, was gradually relaxed. Stage 3 represents the peak period of the epidemic in the empirical area, which was also the turning point of the local epidemic; Due to the traffic control caused by the pandemic and the drastic weakening of people’s willingness to travel, the scale of post-Spring Festival travel rush has shrunk greatly, which made its contribution to the national spread of the epidemic limited. Stage 4 represents the decelerate duration of the local epidemic, and it was at the end of this stage that people in Chinese mainland began to resume work and production in stages. Stage 5 represents the last stage of the local epidemic and the local epidemic was finally controlled.

### 2.3. Methodology

#### 2.3.1. Mapping the Micro-Scale Spatiotemporal COVID-19 Risk

**Figure 3:**
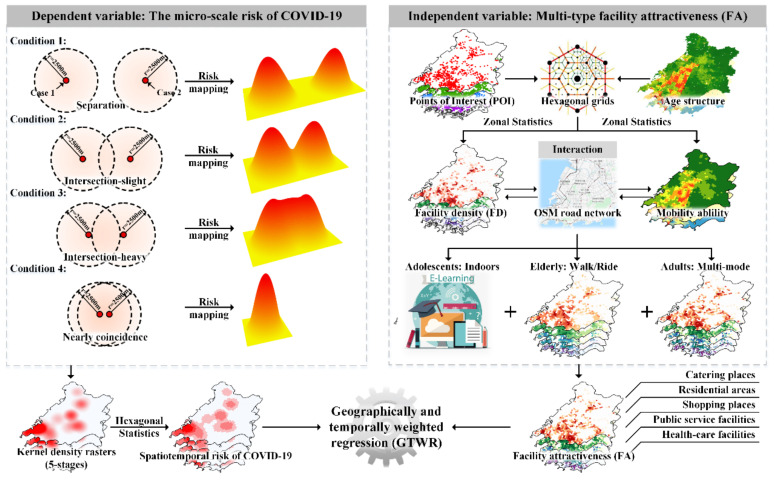
The overall workflow diagram of this empirical modeling study.

Existing theories generally hold that risk decays proportionally with the distance from the source [39], and that the distance decay function it depends on has various forms (such as subsection, gravity, Gaussian, etc.), among which the Gaussian distance decay function has been widely proven to have unique advantages in simulating human travel rules; it is also applicable to the preliminary mapping of epidemic risk [40]. In this study, kernel density estimation (KDE) based on the Gaussian kernel function was used to spatially smooth the epidemic data [41,42]. Kernel density estimation produces a smooth and continuous surface on which each position in the study area is assigned a density value, regardless of any administrative boundary. Considering the space of the empirical area and the spatial range of COVID-19 cases, the bandwidth of the kernel function was set to 2.5 km and the average density values in each grid were calculated as the epidemic risk values (Figure 3).

#### 2.3.2. Facility Attractiveness (FA) and Optimized Gravity Model

The gravity model is a mathematical model that is suitable for the study of human activities; it is an extension of Newton’s law of gravity in the field of social sciences [43,44]. This model indicates that the force between two places is proportional to their “mass” and inversely proportional to the square of the distance between them, and that the “mass” in the model can be replaced by equivalent demographic indicators [45]. In this study, the density of facilities and the number of people in an area were used as modeling indicators. The formula is expressed as follows:

(1) $$FA_{it}=k(FD_{it})\sum_{j}(POP_{j})d_{ij}^{-2}$$

where *FA_it_* is the total attractiveness of facilities *t* in grid *i*; *FD_it_* is the number of facilities *t* in grid *i*—that is, the facility density mentioned above; *POP_j_* is the population (100,000 people) of grid *j*; *d* is the Euclidean distance between grid *I* and grid *j*; *k* is a constant, which is usually set to 1 in practical applications.

Previous literature has proven that the law of human travel within cities is more in accordance with the Gaussian function than with the power function [46,47]. In addition, the definition of “life circle” popularized by the modernized urban planning concept has greatly reduced peopl’s daily travel ranges [48,49]; this is particularly evident in China within the epidemiological context due to the strict community control and lockdown measures imposed [50]. Moreover, different age groups have different travel abilities. Thus, an age-hierarchical Gaussian optimized gravity model was constructed to make it more suitable for use in empirical studies in the context of COVID-19 [51,52]:

(2) $$FA_{it}=(FD_{it})\left[\sum_{j}(POP_{j(20-69)})W_{ij(20-69)}^{}+\sum_{j}(POP_{j(70+)})W_{ij(70+)}^{}\right]$$

(3) $$W_{ij\left(20-69\right)}=\left\{\begin{array}{c} \frac{e^{-\frac{1}{2}}{\left(\frac{t_{ij}}{3600}\right)}^{2}-e^{-\frac{1}{2}}}{1-e^{-\frac{1}{2}}},\;t_{ij}<3600\\ 0,\;t_{ij}\geq 3600 \end{array}\right.;\;W_{ij\left(70+\right)}=\left\{\begin{array}{c} \frac{e^{-\frac{1}{2}}{\left(\frac{s_{ij}}{1200}\right)}^{2}-e^{-\frac{1}{2}}}{1-e^{-\frac{1}{2}}},\;s_{ij}<1200\\ 0,\;s_{ij}\geq 1200 \end{array}\right.$$

The steps used for the construction of the age-hierarchical Gaussian optimized gravity model were as follows (Figure 3):

(1) We carried out a topology inspection and correction on OSM road network data and calculated the mileage *s_ij_* between place *i* and place *j*. On this basis, the hierarchy of the OSM road network was considered as well as the actual situation of Qingdao, the average travel speed of roads was set for all levels, and the travel time *t_ij_* was calculated. These two data points were used to replace the role of *d_ij_* in the traditional gravity model in this study (Table 1).

(2) During the pandemic in the first half of 2020, almost all public transportation was suspended in most parts of China, which made self-driving travel the only realistic and convenient way to travel long distances. Since Chinese laws prohibit citizens under the age of 18 and over the age of 70 from obtaining a motor vehicle driving license, the possible travel modes used by people of different age groups would have been quite different in the pandemic era. In order to take into account the heterogeneity of the mobility and travel range of the age-hierarchical population, the total population was divided into three groups according to their ages: 0–19 years old (adolescent group), 20–69 years old (adult group), and 70+ years old (elderly group). According to the previous references and the research group’s visit to Qingdao [52,53,54,55]:
  - Due to campus closures and the strict community control measures implemented during the first wave of the pandemic, most students (under 20 years old) received their education online and lacked sufficient time or motivation to travel [53]. Therefore, this group, which also had extremely limited travel possibilities, was not included in the model used in this study.
  - As most elderly people over 70 years old do not live with their children in China, this group are likely to have a high travel frequency in order to carry out necessary daily [56]. However, due to the limitations of transportation modes and mobility levels, the range of activities of elderly people is generally limited to less than 1200 m [57,58]. Therefore, in this model we set 1200 m as the travel threshold for the 70+ age group in order to calculate the attractiveness of various facilities to the elderly more reasonably.
  - Qingdao has become one of the cities in China with the longest average travel times due to the separation of occupation areas and residential spaces [59]. The 20–69 age group is the most active group and has the largest travel range. Given the diversity of their travel modes, setting our search threshold according to mileage will lead to great deviations. Therefore, in this study referred to survey results and adopted a travel time of 1 h (3600 s) as the travel threshold for the 20–69 age group.
  - Possible travel routes exceeding the travel threshold of the two age groups mentioned above were not considered in this model.
(3) We replaced the power function distance decay function in (1) with the Gaussian distance decay function corresponding to the travel threshold of each age group.
(4) We then summed the calculation results of the multi-age models.

FAs are integrated variables based on the optimized gravity model. The number of independent variables does not increase when they are used in various models compared with traditional FD predictors, meaning that FA predictors have a wide application range and prospect, which enables them to be tested together with the same highly flexible FDs in different models to compare their advantages and disadvantages. This study will also make use of this advantage of FA predictors to carry out further empirical evaluations of their application efficiency.

#### 2.3.3. Geographically and Temporally Weighted Regression (GTWR)

In order to solve the problem of spatial heterogeneity and autocorrelation when modeling spatial information, the local estimation method represented by geographically weighted regression (GWR) is widely used in empirical research in various fields. The advantage of this method is that it can provide local estimators for each predictor [60]. On this basis, geographically and temporally weighted regression (GTWR), which takes spatiotemporal heterogeneity into account, was proposed [61,62,63]. Compared with the traditional GWR, which can only model in phases when processing data with both spatial and temporal dimensions, this often leads to biased and unsmooth results in time series, while the GTWR framework integrating the temporal autocorrelation of data is more advantageous in spatiotemporal epidemiological modeling [64,65]. It can be expressed as:

(4) $$Y_{i}=\beta_{0}\left(u_{i},v_{i},t_{i}\right)+\sum_{k}\beta_{k}\left(u_{i},v_{i},t_{i}\right)X_{ik}+\varepsilon_{i}$$

where (*u_i_,v_i_,t_i_*) is the spatiotemporal coordinate for observation *i* and *β*(*u_i_,v_i_,t_i_*) is the coefficient of the *k*th independent variable *X_ik_* for observation *i*. GTWR integrates spatial coordinates with temporal coordinates by the following formula, so as to “upgrade” the traditional spatial distance to the spatiotemporal distance to build the weight matrix. Therefore, GTWR is suitable for solving both the spatial and temporal nonstationarity of data:

(5) $${\left(d_{ij}^{ST}\right)}^{2}=\lambda \left[{\left(u_{i}-u_{j}\right)}^{2}+{\left(v_{i}-v_{j}\right)}^{2}\right]+\mu {\left(t_{i}-t_{j}\right)}^{2}$$

where $d_{ij}^{ST}$ is the spatiotemporal distance between observation *i* and *j*; *t_i_* and *t_j_* are the time coordinates of stages *i* and *j* which defined following the epi curve. *λ* and *μ* are the weights for harmonizing the differing units between space and time. The bandwidth of GTWR is selected by the Akaike Information Criterion correction (AICc), which converges to the Akaike Information Criterion (AIC) when the sample size is large enough [66]. 

The model is based on the hypothesis of normal distribution, all independent variables contained in GTWR should pass the statistical significance test under the OLS model framework and there is no serious multicollinearity problem (Table 2). The modeling process is based on the add-in program “Geographically and Temporarily Weighted Regression (GTWR)” for the ArcMap 10.7 software, and the Origin 2021 and ArcGIS Pro software are used to chart and visualize the results in 3D.

## 3. Model Results

Table 3 shows the diagnostic information of model estimation under the OLS, TWR, GWR, and GTWR frameworks with the same independent variables to prove the effect of spatiotemporal information on the improvement in model performance. All independent variables passed the significance level test of the OLS model. Among the four models considered in this paper, the GTWR model outperformed all other models in terms of all diagnostic coefficients. Based on the outstanding model efficiency of the models under the GTWR framework and the spatiotemporal three-dimensional attribute of the empirical data, in this study we use the GTWR framework for micro-scale spatiotemporal COVID-19 risk modeling.

**Figure 4:**
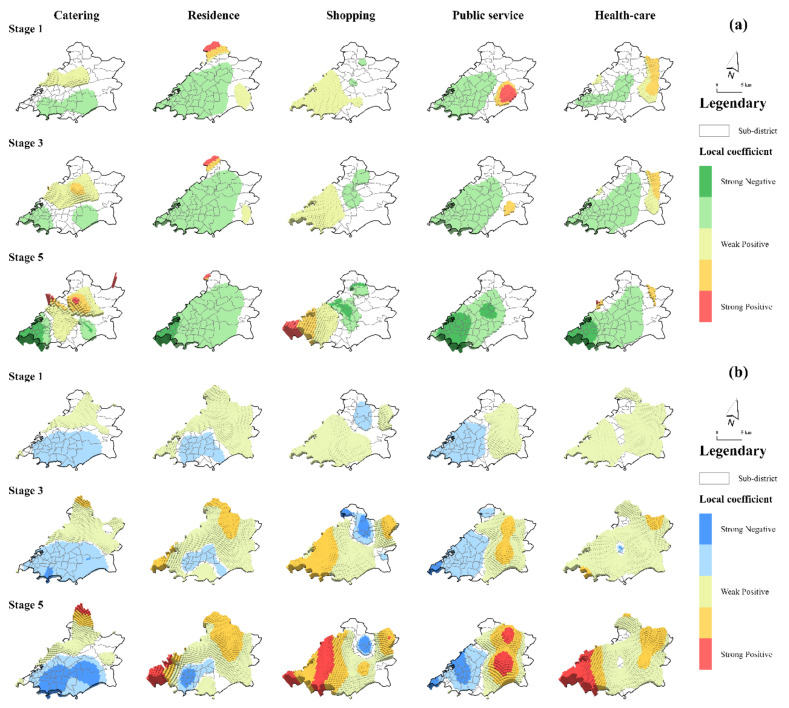
Estimation results for the GTWR coefficients of FD (**a**) and FA (**b**) in various gathering places.

Taking the FD and FA values of five types of gathering places as independent variables, the risk value of COVID-19 in the Qingdao metropolitan area was modeled spatiotemporally and spatiotemporally varying coefficients with local significance levels higher than 95% were visualized (Figure 4). Generally speaking, except for catering places and shopping places, most of the significant GTWR coefficients of FD in various gathering places were negative, demonstrating a negative impact on COVID-19 transmission risk. Residential areas and public service facilities had consistent negative effects in Shibei District, Shinan District, and Licang District from the initial stage of the epidemic, and this effect showed a decreasing trend from the city center to the central fringe. Variables that also showed a strong negative influence in the central city were catering places and health-care facilities. Although the influence of these two variables was not significant in the initial stage, with the transmission of the epidemic they began to have significant effects in the central city. However, shopping places were shown to increase the risk of transmission in the city center during the research period, and this risk increased continuously. Similarly, catering places at the junction of Shibei District and Licang District and public service facilities and health-care facilities in Laoshan District also had remarkable positive effects. 

The result gained using the spatiotemporally varying coefficient of FAs was quite different from the result gained from modeling using FDs as the core variable (Table 4). Almost all the five types of gathering places played a positive role in the spread of the epidemic on a large scale (Figure 4). This promotion was most obvious for the variables of health-care facilities and which most areas were affected, and the intensity of this impact was strengthened with the escalation of risks. The spatiotemporally nonstationary characteristics of catering places and residential areas were similar, showing that Shinan District was the core area exhibiting a restraining effect and Licang District was the core area exhibiting a promoting effect. However, the area affected for catering places was wider than that affected for residential areas and also showed strong restraining characteristics in certain areas of Laoshan District, while the promotion effect of residential areas was more remarkable in Licang District. The coefficients of shopping places and public service facilities showed opposite spatiotemporal characteristics. These coefficients played a significant role in the central city and the edge of the city center, respectively, and the intensity of this role usually reached its peak in the final stage of the epidemic, similar to the modeling results of FDs to a certain extent. 

## 4. Discussion

### 4.1. Comparison of Model Performance between FD and FA

Although, theoretically, facility attractiveness, which integrates the heterogeneity of the regional population distribution, age structure, and facility distribution, is obviously a more suitable indicator for COVID-19 risk modeling than facility density, the theoretical advantages may not be well quantified in empirical studies, especially in different spatiotemporal contexts [16,22,23]. Therefore, we illustrate and discuss the prediction efficiency of FD and FA models at the global and local scales (different risk levels and different administrative regions), respectively, below: 

#### 4.1.1. Global Explanatory Ability of the Models

According to the model diagnostic information for the whole spatiotemporal model, FA is a better predictor for COVID-19 risk modeling than FD (Table 3). An FA model can explain 56.9% of the spatiotemporal risk changes in the empirical area, which is 5.35% more than that explained by the traditional model, and the improvement rate exceeds 10%. At the same time, the AICc value of the model based on the FA predictor is also greatly reduced, which proves that the FA model is more reasonable. 

The cross-sectional explanatory ability of the FA model for spatial COVID-19 risk is also stronger than that of the FD model (Table 5). The predictive ability of the FD and FA models showed an upward trend in almost all the five stages, and the adjusted R^2^ of the FA model reached more than 0.6 at its highest point. 

In addition, in all the five stages, the R^2^ improvement rate of the FA model compared with the FD model was over 10% and was the most obvious (25.0%) in the initial stage of the epidemic, which proved that the effect of “upgrading” FDs to FAs to increase the modeling effectiveness at the initial stage of the epidemic with a limited sample size was remarkable. The reason for this good model performance and the improvement may be that, in the latent and initial stage of the epidemic, due to the extremely limited number of infected persons, it was difficult for these few and scattered people to transmit the virus through short-term contact with crowds in open spaces. This means that areas with a higher facility attractiveness—that is, areas where people are more likely to gather—were crucial places in the spread of the epidemic [29,30]. At this time, due to the lack of virus hosts in the sparsely populated urban fringe areas, the facilities in these areas played a less pivotal role in the transmission of the epidemic [67,68], meaning that the FA predictor had the most obvious optimization effect compared with FD in the initial stage of the epidemic. However, with the spread of virus, there is no such huge shortage of infected people in sparsely populated areas [69]. The magnitude of the role played by facilities in different areas in epidemic transmission tended to be similar, and the optimization effect of the FA predictor declined. However, due to the exponential development of the epidemic [70], FA predictors, which have a larger differentiation in space because of the overlap of the facilities’ density and their effect on causing people to gather, still have advantages over traditional facility densities.

#### 4.1.2. Local Explanatory Ability of Grids with Different Risk Levels

**Figure 5:**
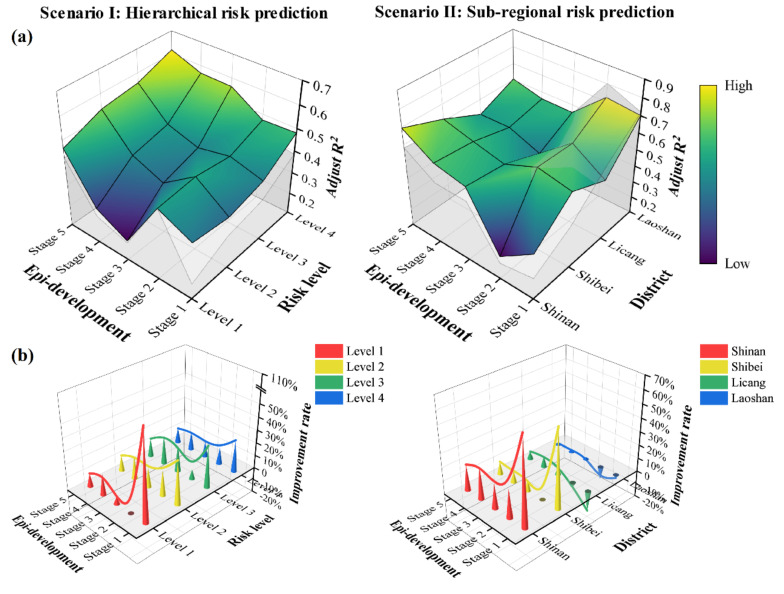
Comparison of the effectiveness of the model prediction between FDs and FAs in two types of application scenarios: (**a**) temporal trend of the effectiveness of the differentiation of the two models (the colored surface is the optimized model, while the gray surface and side filling represent the traditional model); (**b**) improvement rate.

It is necessary to test the prediction ability of the model in grids with different risk levels. The model should be able to accurately identify high-risk grids and avoid overestimating the risk in low-risk areas to the greatest extent in order to prevent unnecessary problems caused by epidemic prevention and control and ensure people’s freedom of travel and the urban vitality in low-risk areas as much as possible. According to the risk value obtained in our Gaussian kernel density analysis, grids were divided into four grades: Level 1 contained grids with a risk value in the top first third among grids with a value greater than 0, Level 2 contained grids with a risk value in the top two thirds among grids with a value greater than 0, Level 3 contained grids with a risk value greater than 0, and Level 4 contained all grids in the empirical area (Figure 5). From the perspective of their ability to predict the risk, both FDs and FAs were relatively stable in low-risk and medium-low-risk areas, being stable between 30% and 60%. Compared with low-risk areas, the stability of the prediction ability of the model in medium-high and high-risk areas, especially in high-risk areas, declined. The temporal stability of the prediction effectiveness was not as good as the performance for low-risk areas, and the risk prediction ability in the initial stage of the epidemic was also limited. From the perspective of improving the efficiency of the model by changing the predictor, the explanatory ability of FA compared with that of the FD model was improved by 10–30% in most conditions, proving that the improvement effect was remarkable. It is worth noting that the prediction ability of the FA model was greatly improved (107.2%) in high-risk areas compared with the FD model in the initial stage of the epidemic, which also shows the significance of population density and age-hierarchical travel capacity in model optimization at the initial stage of the epidemic.

#### 4.1.3. Local Explanatory Ability of Grids in Different Administrative Regions

The optimized model should have the ability to predict the risk of COVID-19 in densely populated areas and economic agglomeration areas in order to maximize socio-economic cost savings. Therefore, the explanatory ability of the model in different administrative regions was compared (Figure 5). The FA model had the strongest ability to predict the risk in Laoshan District, with its ability generally being between 55% and 75%. However, the optimized effect was the least obvious there, and most of the time the model performance was still slightly inferior to that of the traditional model. The fitting efficiencies for Shibei District and Licang District were similar, with both consistently being around 50%, demonstrating a good prediction ability. In the second stage of the epidemic, the FD model was shown to be slightly better than the FA model, but this phenomenon was gradually reversed with the spread of the epidemic. Shinan District is in the old city of Qingdao and also its center; it is a densely populated area and a place where many elderly people live. Although the fitting efficiency of the GIS-based spatiotemporal model in this area is often inferior to that in the other three districts, the effectiveness of the model greatly improved over time from about 40% to about 70%. In addition, the FA model showed the highest level of improvement in this area, which emphasizes the advantages of the optimized method adopted by this research in modeling densely populated areas and aging areas, which are some of the areas most susceptible to epidemics.

### 4.2. Limitation and Prospection

The estimation and comparison results obtained for the models’ performances (global and local fitting effectiveness), as shown above, prove the usefulness of the novel predictor, facility attractiveness (FA), in the spatiotemporal risk modeling of COVID-19 and its superiority over the traditional predictor of “facility density”. This advantage is mainly reflected in the initial stage of the epidemic, as the initial prediction of epidemic risk is often regarded to be weak in previous studies based on the “densities” model [71,72]. Theoretically, the outbreak of a regional epidemic is often started by the entry of a virus carrier in the beginning, and the impact of the effect of gathering on the micro-scale COVID-19 spatiotemporal risk is not obvious at this stage. Models based on facility attractiveness weighted by population density can make up for this deficiency of traditional models [72,73,74]. At the same time, because the limited travel capacity of the elderly means that facilities beyond their travel range cannot be made more “attractive”, the lower FAs value seen in these areas will also separate them from areas where there are more adults who have a stronger travel ability and who are more likely to come into contact with viruses from outside the area, thus reducing the cost of an indiscriminate lockdown [55,58]. In addition, the FA model also has good prediction efficiency for high-risk areas and potential high-risk areas (densely populated areas), which makes it possible to target high-risk areas quickly and accurately at the initial stage of the epidemic. This is beneficial to hierarchical prevention and control strategies for regional epidemics.

However, this model is just the beginning. Limited by the length of this study and the limitation of our empirical area, when constructing the FA predictor, we selectively ignored some other factors that may affect the attractiveness of facilities, such as the complex relationship between the weak travel ability of the elderly and their high possibility of infection [33,55]; the sanitation and disinfection of public facilities [75]; the effect of social distancing and the impact of epidemic prevention measures taken at the individual level, such as wearing masks [76,77]; the different behavior characteristics of human beings from different social classes and with different living conditions within cities [78]; racial differences, which, although almost nonexistent in this empirical area, are very important in Europe and US [46]; factors such as temperature, precipitation and humidity, which some previous studies claim should be considered more and which greatly affect human travel habits within cities [79,80,81]. The construction of FA predictors is not only a formula, but also a reference for thinking. Researchers should combine the characteristics of the study area and modify this model to adapt it to specific local situations. In addition, due to the lack of micro-scale data for the early stage of the epidemic in China’s most epidemic-prone areas, the discussion of this study mainly focuses on the initial stage of the epidemic; the sensitivity and universality of this model need to be supported by subsequent multi-scale and multi-perspective empirical studies. 

Different from traditional models, which require behavior data at the individual level and complicated calculation processes [73,74], the availability of data and strong predictability of multivariate geographic data in COVID-19 risk modeling highlight the need for further spatiotemporal epidemiology research to be carried out, especially research on spatiotemporal risk modeling based on GIS and using geographic methods and spatial analysis techniques [10,12]. More attention should be paid to participation forms and the improvement of the prediction ability of geographic and spatiotemporal analysis methods in applications. This is not only the case for the COVID-19 pandemic but also for the optimization of the regionalized emergency response ability next time human beings have to face a global health crisis.

## 5. Conclusions

This study highlights the importance of considering the heterogeneity of population mobility in order to gain a better understanding of the driving factors spatiotemporally influencing epidemic diffusion in micro-scale areas. Considering this aspect in models will allow us to capture the impact of gathering in various places (the exposure and probability of people gathering) on epidemic transmission more accurately. Therefore, a predictor calculated based on the optimized gravity model, facility attractiveness (FA), is proposed. Geographically and temporally weighted regression is used to measure the effectiveness of this predictor and the spatiotemporal nonstationarity of the influence for the various facilities on epidemic diffusion. The modeling results show that the predictor is superior to the traditional “densities” indicator, especially in areas considered to be high-risk and densely populated during the initial stage of the epidemic. Considering that the novel predictor can only be used with easily accessible data and a relatively simple operation process, it can provide an optimal means for researchers in relevant fields to predict the micro-scale risk of a pandemic.

## Figures and Tables

**Table 1:** Hierarchical road speed setting.

Trip Mode	Road Classification	Speed (km/h)
Walk	Footway, Living Street, Path, Pedestrian, Residential, Service, Steps	5
Drive	Tertiary/Unclassified/Secondary/Primary/Trunk	10/20/30/40/50

**Table 2:** Statistical description of the variables of FD and FA models.

Variables		Characteristic	Mean	Std. Dev.	Min	Max	VIF
Dependent variable	Covid risk	Dynamic	0.06	0.13	0	1.37	-
Independent variables	**Facility density (FD)**						
	Catering	Static	0.75	2.43	0	36	1.92
	Residences	Static	2.24	3.33	0	26	1.62
	Shopping	Static	1.55	5.05	0	80	1.97
	Public services	Static	0.88	1.76	0	13	2.03
	Health-care	Static	0.86	1.95	0	22	1.38
	**Facility attractiveness (FA)**						
	Catering	Static	0.62	2.05	0	30.77	1.94
	Residences	Static	1.83	2.81	0	22.18	1.67
	Shopping	Static	1.29	4.30	0	68.38	1.98
	Public services	Static	0.72	1.47	0	11.25	2.07
	Health-care	Static	0.71	1.62	0	18.78	1.39

**Table 3:** Global diagnostic information for the estimation with FDs and FAs under various regression frameworks.

Diagnostic Information	Facility Density (FD)				Facility Attractiveness (FA)			
	OLS	TWR	GWR	GTWR	OLS	TWR	GWR	GTWR
Adjusted R^2^	0.0827	0.1594	0.4036	0.5159	0.0804	0.1555	0.4078	0.5694
Residual sum of squares	124.84	114.35	81.13	65.86	125.15	114.88	80.56	58.57
AICc	−11,553	−12,258	−15,080	−16,813	−11,531	−12,219	−15,144	−17,690

**Table 4:** Estimation summaries for the GTWR coefficients of the FD and FA models.

Variables	Facility Density (FD)					Facility Attractiveness (FA)				
	Min	LQ	Med	UQ	Max	Min	LQ	Med	UQ	Max
Catering	−10.57	−0.72	0.7	1.32	10.22	−18.33	−4.62	0.4	2.79	38.7
Residence	−63.57	−6.82	−2.61	0.07	4.73	−7.63	0.87	3.39	7.22	25.9
Shopping	−3.21	−0.48	0.11	0.76	9.72	−4.77	0.02	1.71	3.79	11.15
Public service	−16.6	−3.9	−1.44	−0.13	3.37	−11.23	−2.33	1.1	5.86	29.24
Health-care	−19.71	−2.4	−0.57	1.11	6.21	−1.41	2.49	5.2	9.61	40.35
Constant	0	0.02	0.04	0.09	0.53	0	0.01	0.03	0.07	0.42

Note: All coefficients illustrated in Table 4 except for the intercept term need to be multiplied by 10^−3^.

**Table 5:** Phased modeling results of the adjusted R^2^ with FDs and FAs.

Epidemic Stage	Adjusted R2		Improvement Rate
	Facility Density (FD)	Facility Attractiveness (FA)	
Stage 1	0.3847	0.4808	24.99%
Stage 2	0.4190	0.4747	13.28%
Stage 3	0.5039	0.5629	11.72%
Stage 4	0.4950	0.5669	14.51%
Stage 5	0.5532	0.6179	11.70%

## Data Availability

Publicly available datasets were analyzed in this study. The COVID-19 case data can be found in the Qingdao Municipal Health Commission: [wsjkw.qingdao.gov.cn]; The POI data can be found in Amap: [www.amap.com]; The raster data for people of various ages can be found in WorldPop: [www.worldpop.org]; The road network data can be found in OpenStreetMap: [www.openstreetmap.org].
